# Recurrent Neural Network for Predicting Transcription Factor Binding Sites

**DOI:** 10.1038/s41598-018-33321-1

**Published:** 2018-10-15

**Authors:** Zhen Shen, Wenzheng Bao, De-Shuang Huang

**Affiliations:** Institute of Machine Learning and Systems Biology, School of Electronics and Information Engineering, Tongji University, Shanghai, 201804 P. R. China

## Abstract

It is well known that DNA sequence contains a certain amount of transcription factors (TF) binding sites, and only part of them are identified through biological experiments. However, these experiments are expensive and time-consuming. To overcome these problems, some computational methods, based on k-mer features or convolutional neural networks, have been proposed to identify TF binding sites from DNA sequences. Although these methods have good performance, the context information that relates to TF binding sites is still lacking. Research indicates that standard recurrent neural networks (RNN) and its variants have better performance in time-series data compared with other models. In this study, we propose a model, named KEGRU, to identify TF binding sites by combining Bidirectional Gated Recurrent Unit (GRU) network with k-mer embedding. Firstly, DNA sequences are divided into k-mer sequences with a specified length and stride window. And then, we treat each k-mer as a word and pre-trained word representation model though word2vec algorithm. Thirdly, we construct a deep bidirectional GRU model for feature learning and classification. Experimental results have shown that our method has better performance compared with some state-of-the-art methods. Additional experiments about embedding strategy show that k-mer embedding will be helpful to enhance model performance. The robustness of KEGRU is proved by experiments with different k-mer length, stride window and embedding vector dimension.

## Introduction

Transcription factors (TFs) play a critical role in gene expression. It can control genetic information transmission from DNA to messenger RNA by binding to a specific area in DNA sequence^1–3^. The mutations of TF binding sites and its adjacent have great influence on gene expression, and then increase the risk of complex disease^4–8^. It is no doubt that detailed analysis of the TF binding is significant to the further study of gene expression. Many related researches, like digging TF binding sites and exploring the effect of loci mutation on TF binding, have been done in the lab. However, biological experiments for TF binding are expensive and time-consuming. With the development of high-throughput sequencing technology, more and more biological datasets have been proposed^9–11^. Therefore, the principal mission of researchers is to develop a computational model to infer the underlying binding rules and identify TF binding sites without prior information from these datasets.

At the beginning of this study, many computational models, which were used to describe TF binding preference, are proposed based on position weight matrices (PWMs) or motifs^12–18^. These models don’t consider the effect of other sequence features on TF binding, such as low-affinity binding sites, flanking DNA, sequence GC bias, and so on^19–24^. Moreover, ChIP-seq data not only contain TF binding information, but also have the aforementioned sequence features. Therefore, many ChIP-seq-based computational models have been proposed and have better performance than previous models^14,20,25–32^. For example, unlike original kmer-SVM, Mahmoud *et al*.^33^ extracted gapped k-mer feature from ChIP-seq and trained a SVM classifier, which would be used to predict functional genomic regulatory elements and tissue-specific enhancers. This change has greatly improved the prediction performance of original model. In addition, some computational models have made a comprehensive application of ChIP-seq and DNase-seq^27,34–41^, which ensure the prediction accuracy of these models. What’s more, some researchers also proposed related models to identify functional modules from a whole-genome sequence^42,43^, like promoter^44^, enhancer^45,46^, and recombination spots^47^. For instance, a two-layer predictor^45^, named ‘iEnhancer-2L’, was developed to identify enhancers and their strength by pseudo k-tuple nucleotide composition. Liu *et al*.^47^ proposed an ensemble learning approach to identify recombination spots by using multi-modal feature obtained from genome sequence.

Due to the reason that deep learning^48,49^ can learn feature information directly from huge amounts of data, it has developed very rapidly in the past few years and has been widely used in computer vision^50^, image analysis^51–55^, speech recognition^56^, natural language processing (NLP)^57^, and others^58,59^. Recently, researchers used deep learning to extract gene regulation information from DNA sequences^38,60–63^. For example, Babak *et al*.^28^ proposed a model based on deep convolutional neural networks (CNN), named DeepBind, to predict the sequence specificities of DNA- and RNA- binding protein. This model has achieved better performance than other existing methods. Zeng *et al*.^64^ made a systematic exploration of CNN application in DNA-protein binding. The characteristic of this study is that they used a flexible cloud-based framework to achieve the rapid exploration of alternative neural network architectures. These CNN-based models have achieved better performance, but we also note that CNN only focus on the current state and cannot capture the influence of previous state and future state on current state. To address this problem, Quang *et al*.^65^ proposed a hybrid convolutional and recurrent neural network framework for predicting the function of short DNA sequence. Since recurrent neural networks (RNN)^66^ can effectively extract feature information from time-series data, it has been widely used in the process of sequence data, like text classification, video description. In this paper, we use Bidirectional Gated Recurrent Units (GRU)^67^ network to extract feature information from DNA sequence, and then predict TF binding sites by using the feature information.

Neural networks cannot be used for text analysis directly unless we transform text data into the specific format^68–70^. Therefore, word embedding was proposed to solve the defect of one-hot, which can’t reflect the distribution characteristic of text data. For word embedding, word corpus, specific language model and feature learning should be finished at first, and then words or phrases from the corpus are mapped to vectors of real numbers^71–74^. In this paper, k-mer is considered as a word in the sentence, so DNA sequences are divided into a k-mer series with a specified length and stride window. These k-mer datasets would be sent into Bidirectional GRU network for feature learning and classification.

Here, we proposed KEGRU, a novel computational method for predicting DNA-protein binding sties. In KEGRU, a DNA sequence is divided into a k-mer sequence with a specified length and stride window at first. And then, the k-mer sequence is mapped into D-dimensional vector space by word2vec. Thirdly, we use BiGRU to learn features from k-mer sequences and give prediction result. To evaluate the performance of our model, we chose four cell line TF binding datasets HESC, A549, HUVEC and MCF7 from the Encyclopedia of DNA Elements (ENCODE)^75^ project. Experiment results show that our model has better performance than other competing methods on the task of predicting TF binding sites. We prove that k-mer embedding is helpful for the transformation of k-mer sequence. We verify the influence of different k-mer length, slide window and vector length. We also compare the performance of KEGRU with three baseline methods: gkmSVM, DeepBind and CNN_ZH^64^. We hope that our method could contribute to the study of DNA sequence modeling and DNA regulatory mechanisms.

## Results

In this section, we used the Keras platform to implement the KEGRU model. A series of experiments were performed to evaluate the performance of our model. For simplicity, we call a CNN-based model, which was proposed by Zeng *et al*.^64^, as CNN_ZH in this paper. We compared our model with gkmSVM, DeepBind, and CNN_ZH. We evaluated the efficacy of k-mer embedding strategy. We also evaluated the robustness of our model with different k-mer length, stride window, and embedding vector dimension.

AUC (the area under the receiver operating characteristic curve) was used in this paper to evaluate the performance of our model. As a common evaluation metric, AUC is widely used in machine learning and motif discovery. AUC represents a probability, which is generated by a classifier that will rank a randomly chosen positive instance higher than a randomly chosen negative one. In addition, we also used average precision score (APS) to evaluate the performance of our model. APS summarizes a precision-recall curve as the weighted mean of precisions achieved at each threshold, with the increase in recall from the previous threshold used as the weight.

### Experiment setup

To evaluate the performance of our model, we used 125 TF binding sites ChIP-seq experiments from the ENCODE project, including A549, MCF-7, H1-HESC and HUVEC. For each cell type, the centered 101 bps were chosen as positive samples from each record in peak file. To meet model test requirement, equal numbers of negative samples were generated by matching the size, GC-content and repeat fraction of the positive sample. Each dataset was randomly divided into three groups: training, validation and test sets.

For the training of k-mer embedding model, a k-mer corpus was generated by setting *k* to 5, and the stride *s* to 2. We used the python implementation of the word2vec model in Gensim package to obtain the k-mer embedding vectors. All parameters in word2vec were left at their default values.

### Hyper-parameter

The hyper-parameter setting in our methods consists of two groups: model-related and data-related. For model-related, it contains 12 parameter settings, which are generated by the combination of different optimizer and GRU number. Details of the model-related hyper-parameter setting are summarized in Table 1. For each ChIP-seq dataset, we execute all hyper-parameters, and record the performance of different parameter settings and get the best parameter setting by comparing the performance of all datasets. The hyper-parameter of three baseline models, gkmSVM, DeepBind, and CNN_ZH, remain unchanged.

**Table 1:** Hyper-parameter settings.

Hyper-parameter	Optional
GRU units number	50,80,100
Optimizer	SGD, Adam, Adagrad, RMSprop

The whole process is made up of two steps: model training and data statistical analysis. In model training, for each ChIP-seq dataset, we used all hyper-parameters to train KEGRU on the training set with a mini-batch size of 200 and tested it on the validation set. After training, all trained model should be tested on the corresponding test set and record test results. In data statistical analysis, all test results are classified by cell type, hyper-parameter setting and test indicators. The reason for this is that TF may have different binding properties on different cell type and a hyper-parameter setting may have better performance on one cell type and opposite case on other cell types. We would have the best hyper-parameter on specific cell type by comparing the test results.

Although the word embedding has been widely used in NLP, the effect of different embedding strategy on TF binding site prediction is unknown. In addition, we still don’t know the influence of k-mer length, stride window and embedding vector dimension on model performance. Therefore, these settings were also regarded as hyper-parameter and would be discussed later in following section.

### Performance comparison with existing methods

**Figure 1:**
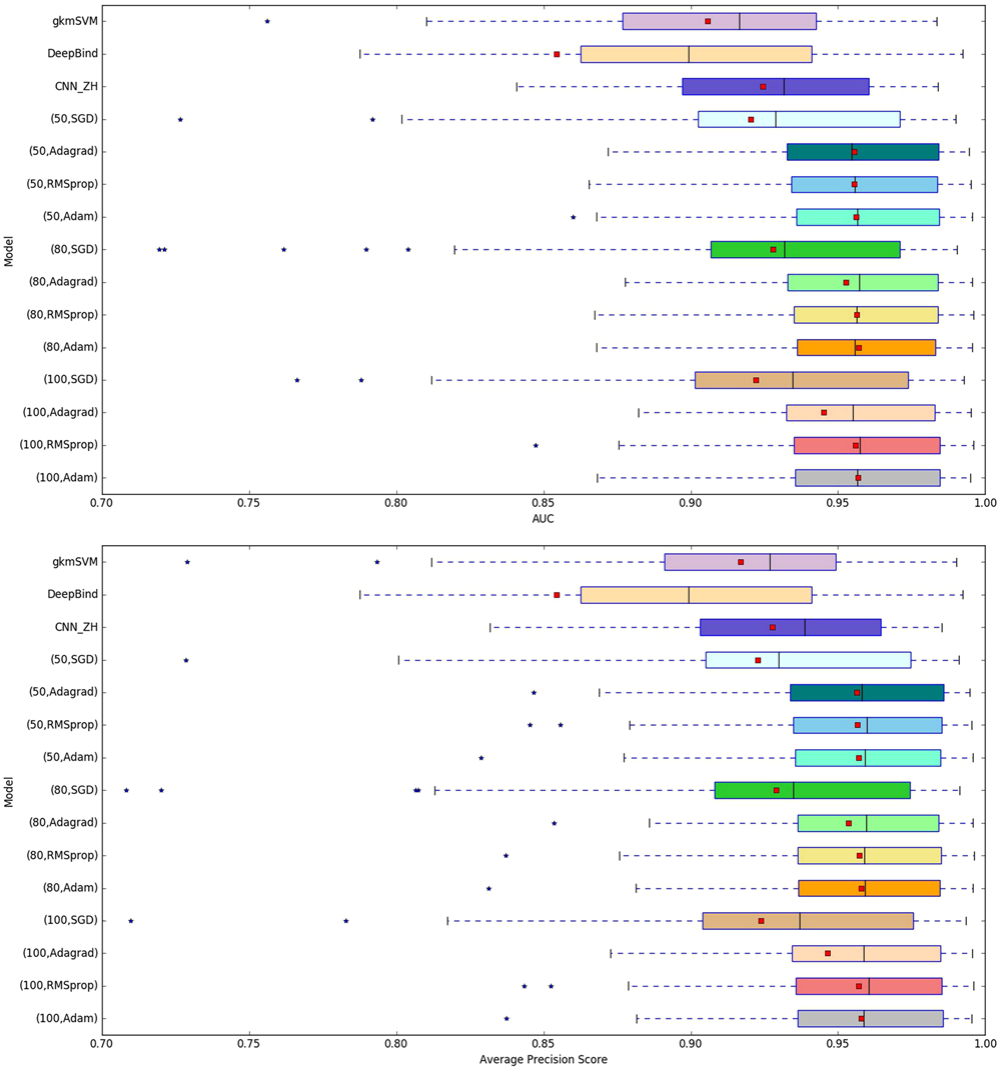
The distribution of AUCs and APSs across 125 experiments in the task of DNA-protein binding prediction.

**Figure 2:**
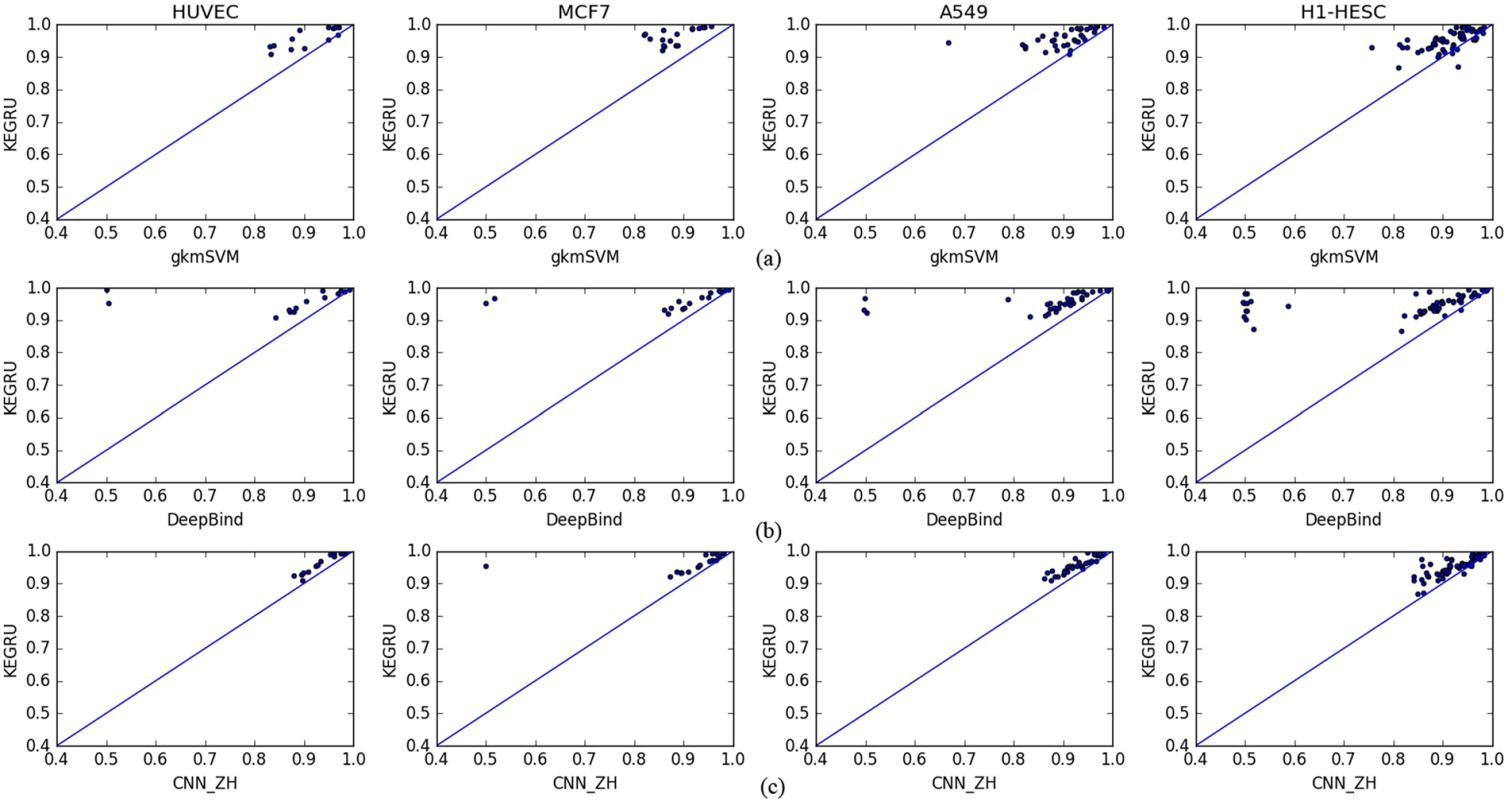
Performance comparison between KEGRU and three baseline models on four cell lines.

To demonstrate the performance of our model KEGRU and compare it with three baseline models gkmSVM, DeepBind and CNN_ZH, we performed a series of experiments with the different hyper-parameter setting. Figure 1 is the concentrated display of the AUC and APS distribution of four models on four cell types with various hyper-parameter settings, highlighting the excellent performance of KEGRU. Table 2 displays the average APS of KEGRU compared with three baseline models. Table 3 displays the average AUCs of KEGRU compared with three baseline models. Given the above information, we chose the best hyper-parameter setting and used scatter plots to display the performance gap of KRGRU and three baseline models. Figure 2(a) shows that KEGRU has higher performance than gkmSVM on four cell line. Figure 2(b,c) show the performance comparison of KEGRU and other two baseline model, respectively. As shown in Fig. 2, our model KEGRU always performs better than three baseline models. In general, with the help of BiGRU and k-mer embedding, our model KEGRU has higher AUC scores than other three baseline models, which means that our model would be helpful for the motif discovery task.

**Table 2:** Average APS scores across 125 ChIP-Seq datasets.

Cell Line	HUVEC	MCF7	A549	H1-HESC
hyper-parm				
gkmSVM	0.9226	0.9016	0.9134	0.9224
DeepBind	0.8601	0.8857	0.8775	0.8291
CNN_ZH	0.9368	0.9173	0.9351	0.9241
50, SGD	0.9423	0.9454	0.9328	0.9046
50, Adagrad	0.9611	0.9648	0.9582	0.9516
50, RMSprop	0.9612	0.9656	0.9601	0.9502
50, Adam	0.9613	0.9656	0.9599	0.9517
80, SGD	0.9429	0.9516	0.9357	0.9144
80, Adagrad	0.9616	0.9657	0.9466	0.9521
80, RMSprop	0.9618	**0.9659**	0.9600	0.9519
80, Adam	0.9618	0.9653	**0.9608**	**0.9528**
100, SGD	0.9424	0.9530	0.9342	0.9041
100, Adagrad	**0.9620**	0.9350	0.9455	0.9469
100, RMSprop	0.9607	0.9655	0.9607	0.9510
100, Adam	0.9619	0.9657	0.9606	0.9525

**Table 3:** Average AUC scores across 125 ChIP-Seq datasets.

Cell Line	HUVEC	MCF7	A549	H1-HESC
hyper-parm				
gkmSVM	0.9119	0.8830	0.9031	0.9129
DeepBind	0.8613	0.8861	0.8784	0.8296
CNN_ZH	0.9335	0.9147	0.9316	0.9210
50, SGD	0.9393	0.9426	0.9285	0.9039
50, Adagrad	0.9603	0.9637	0.9565	0.9509
50, RMSprop	0.9604	0.9646	0.9583	0.9497
50, Adam	0.9605	0.9644	0.9580	0.9513
80, SGD	0.9409	0.9494	0.9320	0.9153
80, Adagrad	0.9610	0.9647	0.9453	0.9514
80, RMSprop	0.9607	**0.9649**	0.9584	0.9512
80, Adam	0.9608	0.9643	**0.9593**	**0.9524**
100, SGD	0.9392	0.9505	0.9301	0.9042
100, Adagrad	**0.9612**	0.9332	0.9431	0.9461
100, RMSprop	0.9599	0.9645	0.9591	0.9502
100, Adam	0.9611	0.9646	0.9588	0.9520

### Evaluate the effect of k-mer embedding

Although word embedding has proven its efficacy in NLP, it is rarely used in human genomic research^76^. The role of the embedding layer in our model is to map the k-mer index to k-mer vectors obtained by the pre-trained k-mer model. Previous experiments have shown that when using k-mer embedding and BiGRU, KEGRU has better performance than other three baseline models. But, the question is that we still don’t know how much the effect of the embedding strategy on model performance is. To address this question, we designed three embedding ways: no-init, init-no-train, and init-train. No-init means that the weights of the embedding layer are initialized with uniform distribution and the neural network can adjust the weights during model training. Init-no-train means that the weights of the embedding layer are initialized by the pre-trained k-mer vectors and will not be updated during model training. Init-train means that the weights of the embedding layer are initialized by the pre-trained k-mer vectors and will be fine-tuned during training. This strategy is adopted in our model KEGRU.

**Figure 3:**
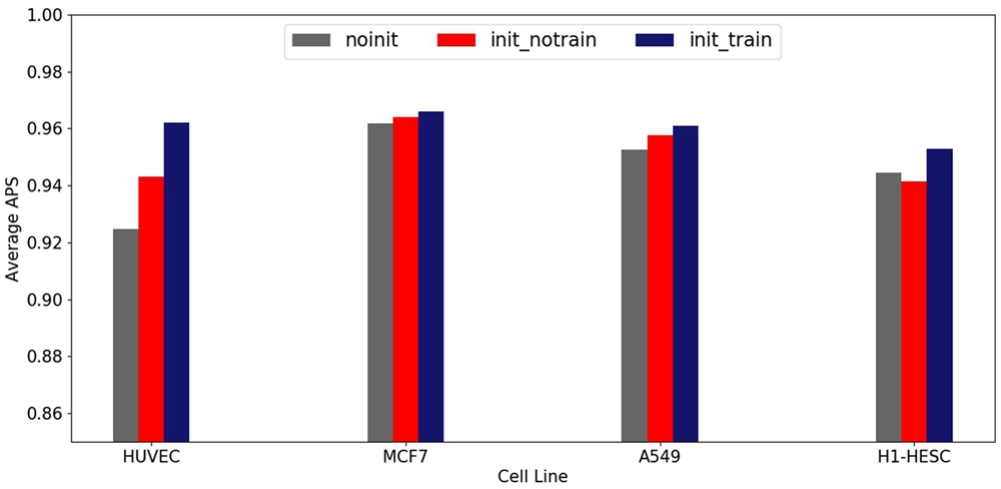
Model performance for different embedding strategies.

Figure 3 displays the average APS of the above three embedding strategies on four datasets. The contrast examination shows that when using pre-trained k-mer vectors, the impact of fine-tuning is minor to the performance of our model. The k-mer embedding strategy has got the desired improvements in the model performance. In conclusion, the pre-trained k-mer vectors could reflect the distribution characteristics of k-mer in DNA sequence well, and help improve model performance.

### Sensitivity analysis

**Figure 4:**
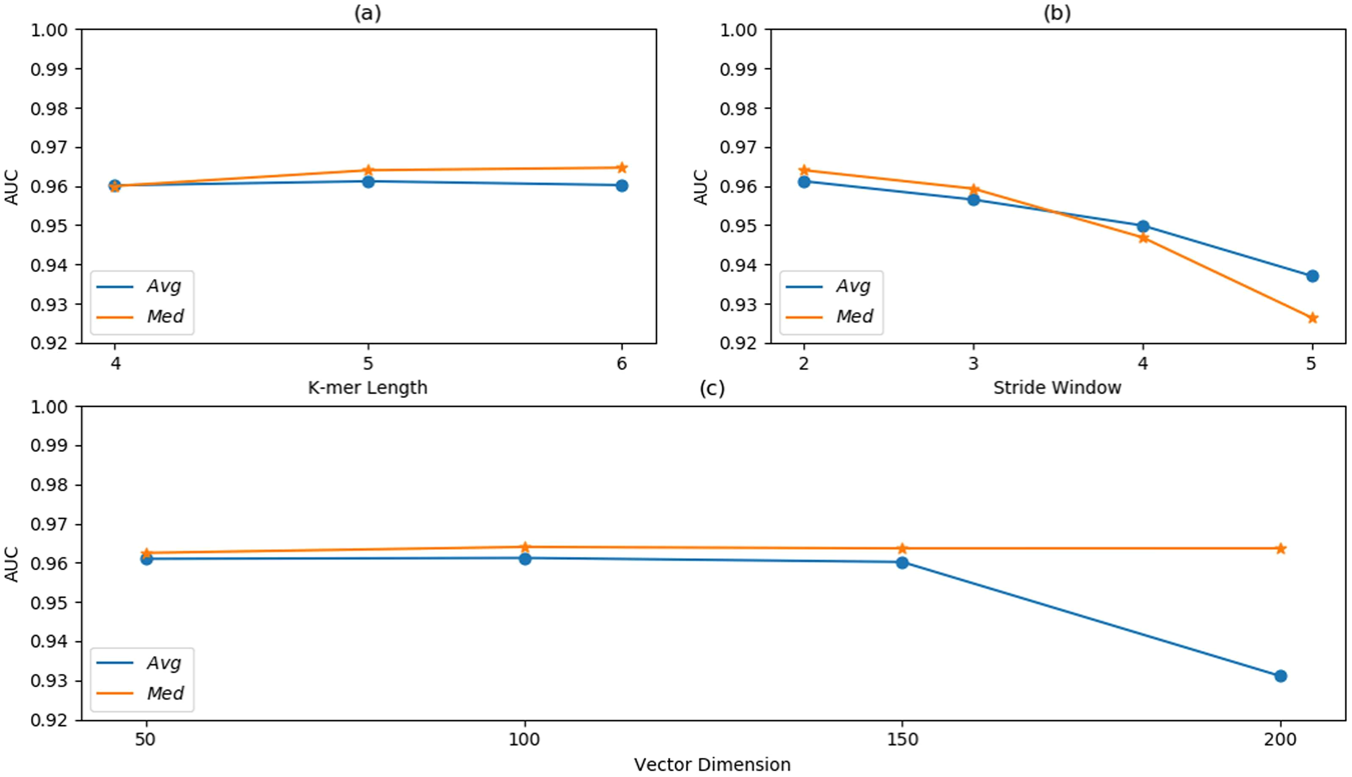
Sensitivity analysis of different k-mer length, stride window and embedding vector dimension, performed on the HUVEC dataset. The average and median AUC scores on HUVEC are reported.

In this section, we used the HUVEC dataset to analyze the effect of k-mer length *k*, stride window *s* and embedding dimension *d*. With the increase of *k*, the capacity of k-mer vocabulary would be explosive growth. Besides, small *k* can’t reflect the characteristics of TF binding. In this paper, we reconstructed the k-mer corpus with different *k* (from 4 to 6) and obtained the k-mer embedding vectors by re-training k-mer model. Figure 4(a) shows that our model has got close prediction performance with different *k*.

The number of k-mer *N* is determined together by DNA sequence length *L*, k-mer length *k* and stride window *s*.

1 $$N=\lfloor (L-k)/s\rfloor +1$$

Figure 4(b) shows that when using four different stride window *s* = 2, 3, 4 and 5, the performance of our model is decreasing. As shown in Equation (1), the size of the k-mer corpus will be decreased by a larger *s*, and can lead to the lack of useful information. This may have negative impact on the embedding representation. We don’t specify the stride window as 1 because it may sharply increase the size of the k-mer corpus and make a k-mer largely overlap with its neighbor. This also has great influence on the embedding representation. In general, to make the embedding layer work well, a proper stride window *s* 2 is recommended here.

In addition, we also analyzed the effect of different embedding dimension *d*, including 50, 100, 150 and 200. The model complexity will be increased by more weight parameters, which is caused by a larger *d* and needs to be learned in embedding layer. As shown in Fig. 4(c), although changes in embedding dimension *d* may lead to overfitting, the prediction accuracy of our model remains fairly constant throughout the experiment.

## Discussion

In this paper, we propose a bidirectional gated recurrent unit neural network with k-mer embedding to identify TF binding sites from DNA sequence. The characteristic of our model is summarized as follows. Firstly, word embedding has been introduced in our model and applied to k-mer sequence representation using the unsupervised learning algorithm word2vec. To avoid dimension curse caused by one-hot encoding, the k-mer embedding vectors are used for feature representation. This measurement is beneficial to following feature learning and classification task. Secondly, our model KEGRU is suitable to processing variable-length input sequences. The shortcoming of CNNs is that the length of input sequence must be fixed. The application of BiGRU network not only improves the compatibility of variable length input sequence, but also is able to capture complex context information from the k-mer sequence. In addition, we prove that our model has better prediction performance than other baseline methods. We also prove that k-mer embedding is an effective method to improve the performance of our model. What’s more, we show the robustness of our model through hyper-parameter experiments. We also show the role of NLP and RNN in the DNA sequence analysis.

There are still some works to be done in the future to improve the performance of our model. First, in the double-stranded DNA sequences, domain-specific modifications may appear identically on one strand or its reverse complement. Therefore, GRU with reverse complement mechanism is helpful to feature learning and classification task. Second, the attention mechanism is also an excellent choice. Attention mechanism has been used in document classification and sentiment classification and achieved better performance. The basic idea of attention mechanism is that it can focus on the key parts of the whole sequence. This characteristic may be used in our model to help explore the important TF binding sites on DNA sequences. In NLP, there are some existing methods to embed sentence or documents into a vector directly by sentence2vec and paragraph2vec. Therefore, we can design a new embedding algorithm for the representation of the variable-length k-mer sequence. Finally, we hope that our method would contribute to the study of gene regulation mechanism.

## Methods

In this section, we describe the basic structure of KEGRU at first. Then, we discuss the detail information of word embedding in our model, which is used to represent a k-mer as a low-dimensional vector. At last, we used Bidirectional GRUs to capture long range dependencies and form fixed-length feature representation of arbitrary-length DNA sequences.

### Model architecture

Given the k-mer length *k* and stride widow *s*, a DNA sequence with *L*_*D*_ base pairs will be split into a k-mer sequence *KS* with length ${L}_{k}=\lfloor ({L}_{D}-k)/s\rfloor +1$. Each k-mer in *KS* is indexed by positive integers in $K=[1,2,\cdots ,N]$. Then, we will explore an appropriate approach to learn the co-occurrence information of k-mer in *KS*, which will help map k-mer sequence into a vector space *V*.

**Figure 5:**
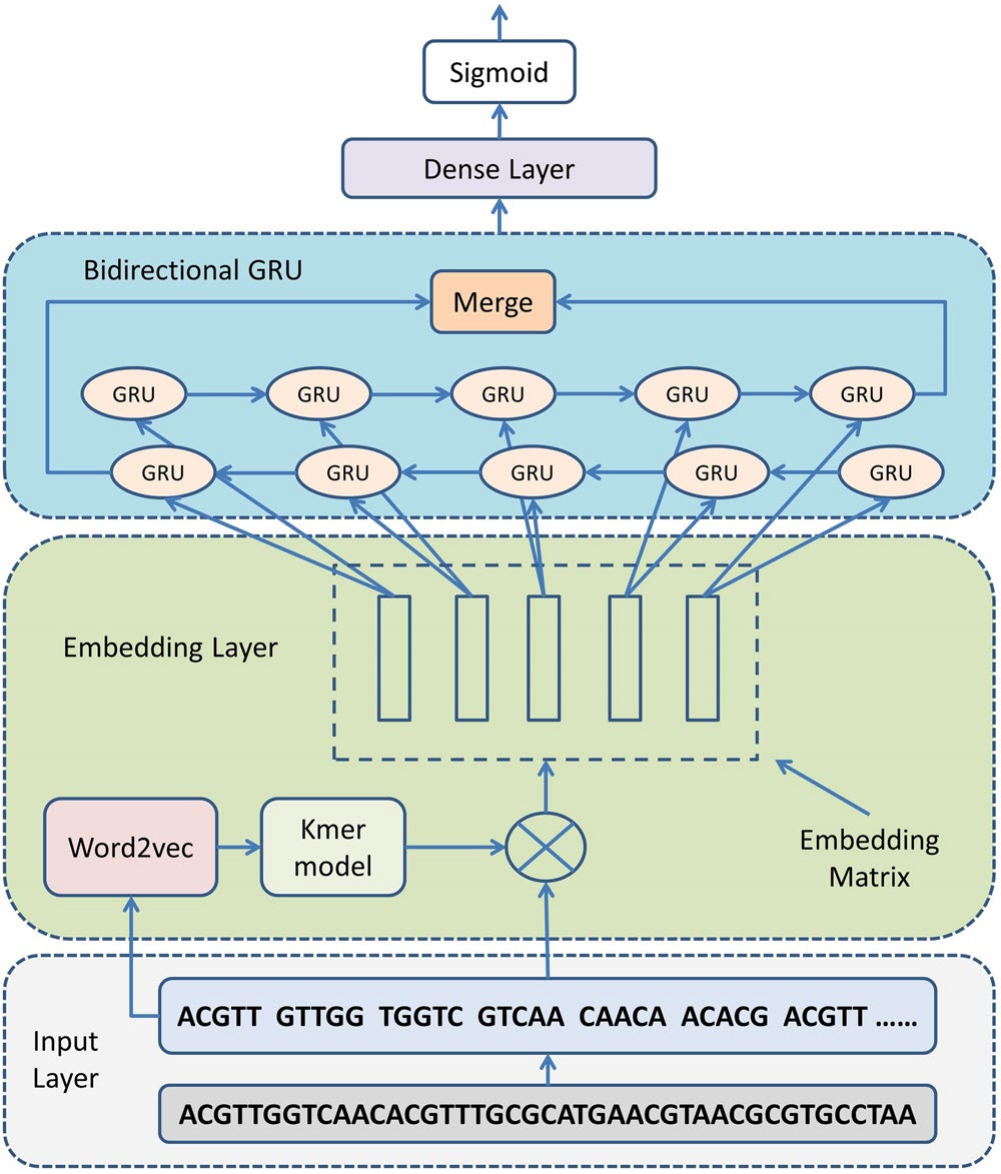
The basic architectural structure of our model KEGRU. (1) We first built the k-mer corpus, which consists of a number of k-mer sequence built by splitting DNA sequence. (2) Based on the k-mer corpus built at first step, we use the pre-trained model word2vec to learn the k-mer embedding vectors. All k-mer vectors are stacked into the embedding matrix that will be used to initialize the embedding layer. (3) We use bidirectional GRU network to solve long-range dependencies problem and to learn feature information from input k-mer sequence. (4) The prediction results were generated by the dense layer and the sigmoid layer, and then we use a loss function to compare the prediction results with the true target labels.

In KEGRU, each DNA sequence is given a binary label, which represents whether the short DNA sequence is a TF binding region or not. Suppose that we have *M* labeled instances ${\{{x}_{i,}{y}_{i}\}}_{i=1}^{N}$, where ${x}_{i}\in {K}^{{L}_{k}}$, ${y}_{i}\in (0,1)$. Our task is to build the prediction model that is used to predict the label for each instance. Figures 5 shows the basic structure of KEGRU. We use formula (2) to represent the entire flow:

2 $$Y={f}_{pred}({f}_{GRU}({f}_{embed}(x)))$$

where *x* denotes a k-mer sequence.

**Figure 6:**
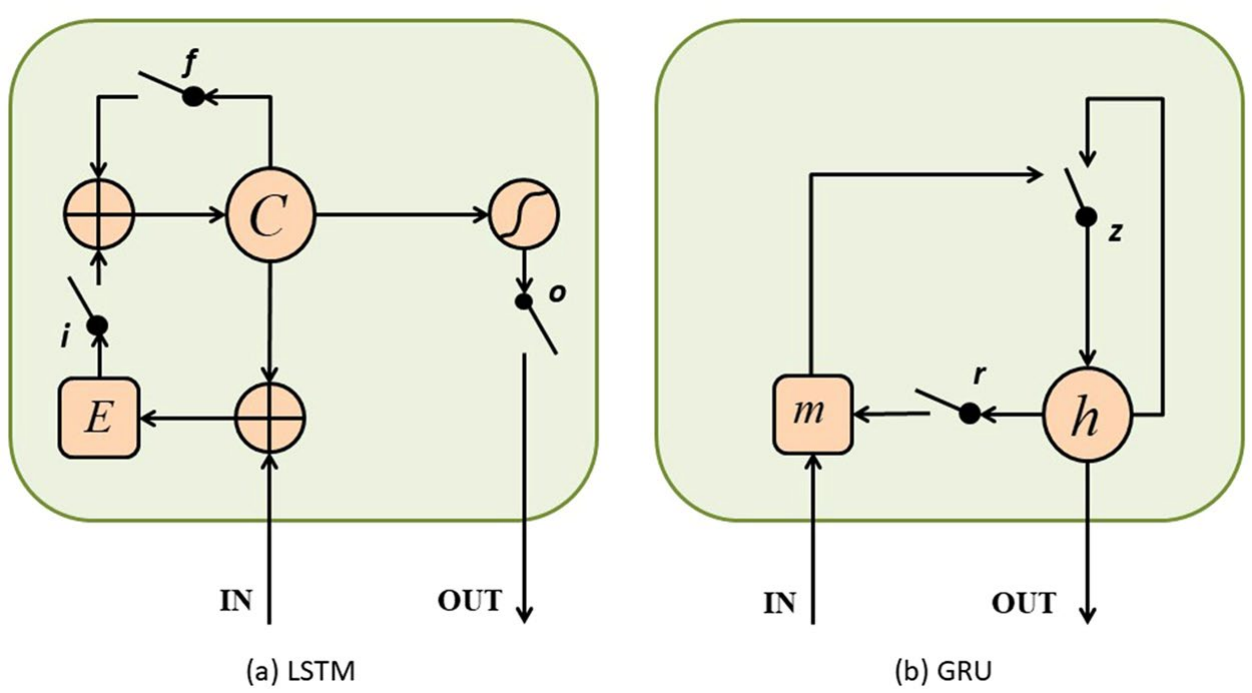
Structural comparison between (**a**) LSTM and (**b**) GRU^67^. (**a**) *i*, *f* and *o* denote the input, forget and output gates, respectively. *C* and *E* denote the current memory cell state and the new memory cell state. (**b**) *r* and *z* represent the reset and update gates. *h* and *m* are the current unit state and the candidate unit state.

During k-mer embedding, a k-mer will be mapped into a vector by learning the co-occurrence statistics of each k-mer in k-mer sequence. We use a Bidirectional GRU network to capture the long-term dependencies, and then generate a fixed-length feature vector. In the prediction stage, we will obtain a prediction by performing a logistic regression on the feature representations. Given k-mer sequence *x*_*i*_ and model parameters Φ, the conditional likelihood of predicting label *y*_*i*_ is computed by^76^:

3 $$\mathrm{log}\,p({y}_{i}|{x}_{i},{\rm{\Phi }})={y}_{i}\,\mathrm{log}\,\delta ({\beta }^{T}{h}_{i})+({\rm{1}}-{{\rm{y}}}_{{\rm{i}}}\,\mathrm{log}(1-\delta ({\beta }^{T}{h}_{i})))$$

where *β* denotes the prediction parameter. *h*_*i*_ represents the learned fixed-length feature. *δ* denotes the logistic sigmoid function. Our model is trained by minimizing the loss function:

4 $$\phi =-\,\sum _{i=1}^{N}\mathrm{log}\,p({y}_{i}|{x}_{i},{\rm{\Phi }})$$

### K-mer embedding with Word2vec

If we use one-hot^77^ to encode each word in a large number of text data, the word vector may be a high-dimensional vector. We have to consume more and more computer resources to store and process these vectors. To address this problem, researchers proposed the concept of distributed representation^78–81^, including word embedding. Briefly, the core idea behind word embedding is to model and analyze semantic similarities between words based on their distributional properties in large samples of document data. After word embedding, words from the corpus would be mapped to vectors of real numbers, which can be processed by a neural network model.

In practical use, all the word vectors will be deposited into a matrix $WE\in {R}^{d\times N}$, where *N* denotes the size of the corpus and *d* denotes the word vector dimension. We call this matrix as the embedding layer or the lookup table layer. The embedding layer can be initialized through a pre-trained algorithm, and some algorithms have been proposed based on neural networks^70^, dimensionality reduction on the word co-occurrence matrix^72^, probabilistic models^82^, and explicit representation in terms of the context in which words appear^83^. For example, word2vec is a set of related models based on continuous bag-of-words and skip-gram. These models are sample neural networks that are trained to generate the context information of the word. As an unsupervised learning algorithm, Glove was used to learn word feature information from a corpus. Word vector is obtained through global word-word co-occurrence statistics.

In our model, each k-mer in k-mer sequence is considered as a word in the sentence. Therefore, we can use word embedding to represent a k-mer sequence at the word level. Given a k-mer sequence *KS* consisting of N k-mers, it can be represented as $KS=\{{k}_{1},{k}_{2},{k}_{3},\,\cdots ,\,{k}_{N}\}$. We first train a k-mer model *KM* through word2vec. Then, the vector of k-mer *k*_*t*_ is obtained by *KM*:

5 $$k{v}_{t}=KM({k}_{t})$$

Then, the k-mer sequence *KS* can be represented as $K{S}_{e}=\{k{v}_{1},\,k{v}_{2},\,k{v}_{3},\,\cdots ,\,k{v}_{N}\}$.

### Bidirectional GRU

In order to deal with several shortcomings about the standard RNN model, a list of efforts, including Long short-term memory (LSTM)^84,85^ and other similar approaches, have been proposed in this field. The GRU was proposed by Cho *et al*.^86^. Figure 6 shows the internal structure of LSTM and GRU. In a gated recurrent neural network, each unit can control the flow of information through resetting gate and updating gate, and all memory contents are fully exposed at each time step. Besides, the output of GRU is to achieve a balance between the previous memory state and the new candidate memory state.

The update gate *z*_*t*_ is computed by^67^

6 $${z}_{t}=sigmoid({W}_{z}{x}_{t}+{U}_{z}{h}_{t-1}+{b}_{z})$$

where *x*_*t*_ is the input vector of the GRU. *h*_*t*−1_ is the previous output of the GRU. *W*_*z*_, *U*_*z*_ and *b*_*z*_ are forward matrices, recurrent matrices and biases for update gate, respectively.

Similarly to the update gate, the reset gate is computed by^67^

7 $${r}_{t}=sigmoid({W}_{r}{x}_{t}+{U}_{r}{h}_{t-1}+{b}_{r})$$

where the parameters are as above.

And then, the candidate memory state *m*_*t*_ is computed by^67^

8 $${m}_{t}=\,\tanh ({W}_{h}{x}_{t}+{U}_{h}({r}_{t}\ast {h}_{t-1})+{b}_{h})$$

where *σ*_*h*_ is the hyperbolic tangent function. * is an element-wise multiplication.

Finally, the memory state *h*_*t*_ of the GRU is computed by^67^

9 $${h}_{t}=(1-{z}_{t}){h}_{t-1}+{z}_{t}{m}_{t}$$

To make our model have a flexible input data format and can reach future input information from the current state, we used Bidirectional RNN (BiRNN). The basic idea of BiRNN is that all neurons in regular RNN are split into forward layer and backward layer, which represent the positive time direction and negative time direction, respectively. By using this structure, it is easy to capture the effect of input information from the past and future on current state. The output of BiRNN is computed by merging forward layer out and backward layer out with specific mode, like concatenate, sum, average and multiplication.

In our model, standard RNN unit is replaced by GRU. The output of BiGRU is calculated by

10 $$output=merge(fout,\,bout)$$

where *fout* is the output of forward layer. *bout* is the output of backward layer.

## Data Availability

The datasets generated during and analyzed during the current study are available from the corresponding author on reasonable request.
